# Neurofilament light chain in the vitreous humor of the eye

**DOI:** 10.1186/s13195-020-00677-4

**Published:** 2020-09-17

**Authors:** Manju L. Subramanian, Viha Vig, Jaeyoon Chung, Marissa G. Fiorello, Weiming Xia, Henrik Zetterberg, Kaj Blennow, Madeleine Zetterberg, Farah Shareef, Nicole H. Siegel, Steven Ness, Gyungah R. Jun, Thor D. Stein

**Affiliations:** 1 Department of Ophthalmology, Boston Medical Center, Boston University School of Medicine, 85 E Concord St. #8813, Boston, MA 02118 USA; 2 Department of Medicine (Biomedical Genetics Section), Boston University School of Medicine, Boston, MA USA; 3 Department of Pharmacology and Experimental Therapeutics, Boston University School of Medicine, Boston, MA USA; 4 Geriatric Research Education and Clinical Center, Bedford Veterans Affairs Medical Center, Bedford, MA USA; 5 Department of Psychiatry and Neurochemistry at Institute of Neuroscience and Physiology, Sahlgrenska Academy at University of Gothenburg, Gothenburg, Sweden; 6 Clinical Neurochemistry Laboratory, Sahlgrenska University Hospital, Mölndal, Sweden; 7 Department of Neurodegenerative Diseases, UCL Institute of Neurology, London, UK; 8 UK Dementia Research Institute at UCL, London, UK; 9 Department of Clinical Neuroscience at Institute of Neuroscience and Physiology, Sahlgrenska Academy at University of Gothenburg, Gothenburg, Sweden; 10 Department of Ophthalmology, University of Illinois at Chicago School of Medicine, Chicago, IL USA; 11 Boston University Alzheimer’s Disease and CTE Center, Boston University School of Medicine, Boston, MA USA; 12 Department of Pathology and Laboratory Medicine, Boston Medical Center, Boston University School of Medicine, Boston, MA USA; 13 Department of Veterans Affairs Medical Center, VA Boston Healthcare System, Boston, MA USA

**Keywords:** Alzheimer’s disease, Vitreous humor, Neuro-filament light chain, Ocular biomarkers, Amyloid beta, Tau

## Abstract

**Background:**

Neurofilament light chain (NfL) is a promising biomarker of neurodegeneration in the cerebrospinal fluid and blood. This study investigated the presence of NfL in the vitreous humor and its associations with amyloid beta, tau, inflammatory cytokines and vascular proteins, apolipoprotein E (*APOE*) genotypes, Mini-Mental State Examination (MMSE) scores, systemic disease, and ophthalmic diseases.

**Methods:**

This is a single-site, prospective, cross-sectional cohort study. Undiluted vitreous fluid (0.5–1.0 mL) was aspirated during vitrectomy, and whole blood was drawn for APOE genotyping. NfL, amyloid beta (Aβ), total Tau (t-Tau), phosphorylated Tau (p-Tau181), inflammatory cytokines, chemokines, and vascular proteins in the vitreous were quantitatively measured by immunoassay. The main outcome measures were the detection of NfL levels in the vitreous humor and its associations with the aforementioned proteins. Linear regression was used to test the associations of NfL with other proteins, *APOE* genotypes, MMSE scores, and ophthalmic and systemic diseases after adjustment for age, sex, education level, and other eye diseases.

**Results:**

NfL was detected in all 77 vitreous samples. NfL was not found to be associated with ophthalmic conditions, APOE genotypes, MMSE scores, or systemic disease (*p* > 0.05). NfL levels were positively associated with increased vitreous levels of Aβ_40_ (*p* = 7.7 × 10^−5^), Aβ_42_ (*p* = 2.8 × 10^−4^), and t-tau (*p* = 5.5 × 10^−7^), but not with p-tau181 (*p* = 0.53). NfL also had significant associations with inflammatory cytokines such as interleukin-15 (IL-15, *p* = 5.3 × 10^−4^), IL-16 (*p* = 2.2 × 10^−4^), monocyte chemoattractant protein-1 (MCP1, *p* = 4.1 × 10^−4^), and vascular proteins such as vascular endothelial growth factor receptor-1 (VEGFR1, *p* = 2.9 × 10^−6^), Vegf-C (*p* = 8.6 × 10^−6^), vascular cell adhesion molecule-1 (VCAM-1, *p* = 5.0 × 10^−4^), Tie-2 (*p* = 6.3 × 10^−4^), and intracellular adhesion molecular-1 (ICAM-1, *p* = 1.6 × 10^−4^).

**Conclusion:**

NfL is detectable in the vitreous humor of the eye and significantly associated with amyloid beta, t-tau, and select inflammatory and vascular proteins in the vitreous. Additionally, NfL was not associated with patients’ clinical eye condition. Our results serve as a foundation for further investigation of NfL in the ocular fluids to inform us about the potential utility of its presence in the eye.

## Background

Neurodegenerative diseases have risen in prevalence over the last few decades with the growth of an aging population in Western society [1]. Of the two most common neurodegenerative diseases, Alzheimer’s disease (AD) alone affects 5.5 million Americans while Parkinson’s disease (PD) now affects over 1 million individuals in the USA [2]. Although the diagnosis of neurodegenerative diseases is often based on clinical presentation supported by diagnostic testing during later stages, early diagnosis remains a challenge. There is a need for reliable biomarkers that can serve as a mechanism for early diagnosis, prognostic assessment, and measurable response to treatment for AD and other neurological disorders. More recently, neurofilament light chain (NfL), a promising biomarker of neurodegeneration, has been identified in the cerebrospinal fluid (CSF) and blood as a potential screening tool and prognostic indicator, and it may now be on the brink of clinical applicability [3].

NfL is a subunit of neurofilaments (Nfs), which are proteins exclusively located in the neuronal cytoplasm to provide structural stability and maintain the integrity of neurons and speed of impulses. Large axons need and express both NfL and Nfs. Under normal circumstances, NfL is persistently released at low levels, and with increasing age, higher levels are seen in older individuals [4]. Higher levels of NfL are also seen in both the cerebrospinal fluid (CSF) and blood in those with neurodegenerative, inflammatory, or vascular injury of neurons, in several different neurological diseases, including multiple sclerosis (MS), AD, frontotemporal dementia (FTD), amyotrophic lateral sclerosis (ALS), atypical Parkinsonian disorders (APDs), and traumatic brain injury (TBI). In addition to other biomarkers such as amyloid beta (Aβ) and tau protein, elevated levels of NfL in both the CSF and blood have been shown to differentiate healthy controls from subjects with AD with reliable accuracy [3, 5–9]. Although NfL is not yet being used as a screening tool in the clinical setting, the ability of serum NfL to detect and differentiate people suffering from neurodegenerative diseases from healthy controls has made it an important research subject in the last few decades [10].

The eye is a window to the brain, and they both share a common embryological origin and vasculature [11–17]. Eye diseases such as cataracts, glaucoma, macular degeneration, and diabetic retinopathy have been associated with AD in epidemiological studies, indicating that eye disease and AD possibly share common risk factors and pathological mechanisms at the molecular level [18–22]. The interconnections between the eye and the brain suggest that elucidating the common features of neurodegenerative processes can lead to a better understanding of both neurological and eye diseases [23].

Recently, we found that cognitive function, measured by Mini-Mental Status Examination (MMSE), in patients with eye disease is significantly associated with the levels of amyloid β (Aβ_40_ and Aβ_42_) and total tau (t-tau) proteins in the vitreous humor [24]. We now hypothesize that NfL can be identified in the vitreous and associated with other relevant biomarkers of neuronal origin.

## Methods

This was a prospective, cross-sectional cohort study conducted at Boston University Medical Center (BUMC). Approval and oversight for the study protocol were provided by the BUMC Institutional Review Board (study reference number H33883) and was in accordance with the ethical standards of the Committee on Human Experimentation of our institution and the Declaration of Helsinki.

The aim of this study was to detect the presence of NfL in the vitreous humor of the eye and assess its associations with core biomarkers for AD (Aβ_40_, Aβ_42_, t-tau, and p-tau181), inflammatory and vascular proteins, MMSE scores, apolipoprotein E (APOE) allele status, and ophthalmic and systemic diseases.

Vitreous humor, a gelatinous non-regenerative fluid that fills the posterior segment of the eye, is accessible by needle aspiration in the office setting or by surgery in the operative setting. Study participants were selected based on the following criteria: age 18 years or older, primary language English or Spanish, and those scheduled for pars plana vitrectomy in at least one eye for a clinical eye condition. Surgical indications for vitrectomy included various vitreoretinal disorders, such as rhegmatogenous retinal detachment (R-RD), macular hole (MH), epiretinal membrane (ERM), or complications of diabetic retinopathy (DR) such as vitreous hemorrhage and tractional retinal detachment. Written informed consent was obtained from all patients who participated in the study, and no patients were excluded due to existing ocular or medical comorbidities.

Demographic and clinical data were obtained from patients once enrolled through the completion of a patient questionnaire as well as access to those patients’ electronic medical records. Demographic data included race and the highest educational level completed, along with athletic and military history. Clinical information was collected on study participants, including medical and smoking history, history of head and/or neck injuries, family history of cognitive dysfunction, and subjective cognitive complaints. Furthermore, patients’ baseline color vision, ocular history, and family history of ocular disease were obtained. All study participants were administered an MMSE within 1 week prior to their eye surgery.

### Biospecimen collection

Vitreous samples were collected at the start of each vitrectomy procedure by the surgical team, and 0.5–1.0 mL of undiluted vitreous fluid was aspirated via the vitrectomy probe into an attached sterile 3-mL syringe. Infusion of saline into the vitreous cavity was immediately undertaken in order to re-pressurize the posterior chamber of the eye. The syringe containing the specimen was then capped using a sterile technique and directly handed to a research assistant who labeled it with a pre-determined non-identifiable study number and placed the sample on ice for transport. In the Molecular Genetics Core Laboratory (MGCL) at Boston University, the vitreous samples were aliquoted into 900-μl Eppendorf tubes and stored at − 80 °C until analysis. Aside from the collection of the vitreous samples, each study participant’s vitrectomy was completed according to the clinical standard of care for that patient’s ocular condition.

Blood samples were collected on a separate occasion from all study participants prior to surgery. Eighteen milliliters of whole blood was drawn from each patient into EDTA-treated purple top tubes. The MGCL processed the de-identified blood samples into their component serum, plasma, and buffy coat. The buffy coat was later used for DNA extraction and *APOE* genotyping.

### Immunoassay measurements for neurofilament light chain

Vitreous samples were sent to the Institute of Neuroscience and Physiology, Sahlgrenska Academy at the University of Gothenburg in Sweden. Vitreous fluid NfL concentration was measured using the commercially available NF-Light kit on a single-molecule array (Simoa) HD-X Analyzer (Quanterix, Billerica, MA) in one round of experiments using one batch of reagents. Coefficients of variation for QC samples at concentrations of 8.8 and 149 pg/mL were 2.1% and 8.9%, respectively.

### Immunoassay measurement for amyloid, tau, and inflammatory cytokines, chemokines, and vascular proteins

Vitreous samples were centrifuged for 15 min at 12,000 rpm to separate the cellular contents, aliquoted at 100 μl, frozen at − 80 °C, and then used for Aβ, t-tau, and p-tau181 measurements. Briefly, assays were run per the manufacturer’s instructions and in duplicate for beta-amyloid 1–40 and 1–42 (Meso Scale Discovery (MSD), Rockville, MD, #K15200E-2), tau phosphorylated at threonine 181 (p-tau181), and t-tau (MSD #K15121D-2), using capturing antibody AT270 against p-tau181 (Thermo Scientific #MN1050) and T46 antibody against total tau as the detecting antibody (Thermo Scientific). Neuroinflammatory cytokines were measured using Neuroinflammation Panel 1 (K15210G, MSD). Samples were diluted 1:2 for pro-inflammatory panel 1 (IFN-γ, IL-10, IL-12p70, IL-13, IL-1β, IL-2, IL-4, IL-6, IL-8, TNF-α), cytokine panel 1 (IL-12/IL-23p40, IL-15, IL-16, IL-17A, IL-1α, IL-5, IL-7, TNF-β, VEGF-A), and angiogenesis panel 1 (basic FGF, VEGFR-1/Flt-1, Tie-2, VEGF-C, VEGF-D), or 1:5 for vascular injury panel 2 (SAA, CRP, VCAM-1, ICAM-1). Sulfo-tag-conjugated anti-mouse secondary antibody (MSD) was used for signal detection by the MSD platform, and an MSD SECTOR S 600 Imager was used to measure the analyte levels.

### Apolipoprotein E genotyping

DNA was extracted from the buffy coat, and single nucleotide polymorphisms from the *APOE* gene (National Center for Biotechnology Information SNPs rs429358 and rs7412) were examined using TaqMan assays (Applied Biosystems, Foster City, CA).

### Statistical analysis

Due to the skewed distributions of the levels of NfL and other biomarkers, the protein levels were log-transformed after adding one. We performed association tests on vitreous NfL with vascular/inflammatory-related biomarkers, also in the vitreous, as quantitative outcomes under a linear regression model. We assessed the effects of *APOE* genotypes (ε2 and ε4), used as predictors, with NfL levels as well as other biomarkers as quantitative outcomes, also using linear regression, with gender and age at the time of the eye exam as covariates. In addition, we conducted an association of NfL levels with MMSE test scores as a quantitative outcome, adjusting for gender, age, and education level as covariates. A term for education level was categorized into three levels, including (1) less than or equal to 8th grade level, (2) 9th grade to high school, and (3) greater than or equal to college (bachelors or associates). The covariate education level was analyzed as an ordinal and categorical variable. *p* values of less than 0.05 were regarded as statistically significant. Because patients with diabetes are at higher risk for the development of dementia and AD as well as retinal damage in the eye, we sought to determine vitreous associations independent of diabetes, so our results were adjusted for diabetes. To account for the multiple cytokines and vascular proteins tested, the Bonferroni correction was conducted to determine which ones remained statistically significant. The number of analytes corrected for includes all the proteins tested in this study (*n* = 41), including NfL, resulting in a new *p* value cutoff of 0.0012. Additionally, due to our highly diverse patient cohort, NfL levels were compared to race and ethnicity by means of the analysis of variance (ANOVA).

## Results

**Fig. 1:**
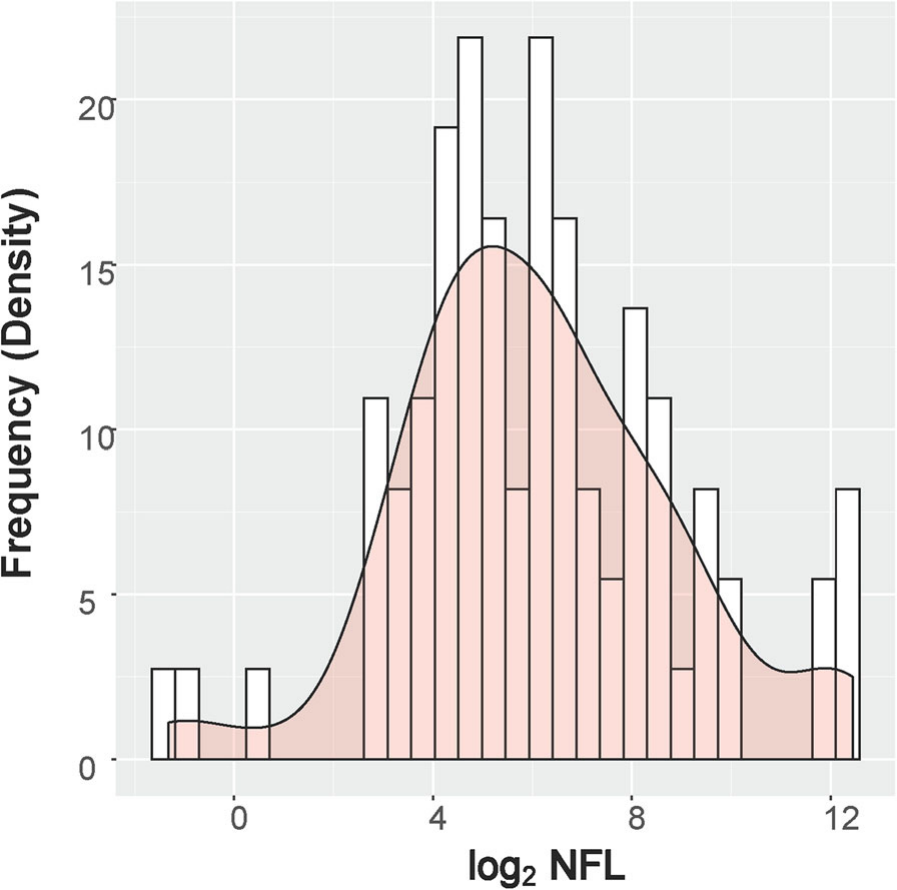
Normal distribution of NfL. Distribution of neurofilament light chain levels in the vitreous humor after log_2_ transformation

The results identified NfL protein in the vitreous humor of all 77 samples from 77 unique subjects. Sixty-three percent of the subjects were male, and the mean age was 56.2 years. The ethnic breakdown of our sample population is a close representation of the patient population typically seen at the eye clinic at BUMC, and 69% of the sample population were over the age of 50 years (Table 1). The data indicates there were no structural biases in the sample population among disease category, age, gender, race, or educational level. Of the 77 subjects, 55 (71.42% of the cohort) had 20 pg/mL or more NfL protein present in the vitreous humor. The median NfL level was 68.65 pg/mL, and the mean level was 432.67 pg/mL ± 1124.47 pg/mL, a large difference due to the skewed distribution in the raw data. But after the NfL values were transformed via quality control and log_2_ transformation, NfL proteins were found to have a normal distribution in the vitreous humor (Fig. 1).

**Table 1:** Patient demographics and overall sample breakdown

*Characteristic*	*Overall sample breakdown (N = 77)*
Sex	
Male	49 (63.63%)
Female	28 (36.36%)
Age	
Mean age ± SD	56.21 ± 15.52
Age > 40	68 (88.31%)
Age > 50	53 (68.83%)
Age > 60	35 (45.45%)
Race	
Asian	3 (3.90%)
American Indian or Alaskan Native	2 (2.60%)
Black or African	27 (35.06%)
Hispanic	11 (14.29%)
White	26 (33.77%)
Others	8 (10.39%)
Ethnicity	
Hispanic	20 (25.97%)
Not Hispanic	57 (74.03%)
Education level	
8th grade or less	11 (14.29%)
High school	28 (36.36%)
College or greater	38 (49.35%)
Eye disease category	
Age-related macular degeneration	4 (5.19%)
Diabetic retinopathy	35 (45.45%)
Age-related cataracts	66 (85.71%)
Glaucoma	10 (12.99%)
Epiretinal membrane	17 (22.08%)
Retinal detachment	32 (41.56%)
Macular hole	12 (15.58%)
APOE allele	**Overall sample breakdown (*n*** **= 76, 152 alleles)**
ε2 (may offer some protection against AD)	15 (9.87%)
ε3 (neutral risk for AD)	121 (79.61%)
ε4 (increases risk for AD at an earlier age)	16 (10.52%)
APOE genotype	**Overall sample breakdown (*n*** **= 77)**
ε22	0 (0.0%)
ε23	11 (14.29%)
ε24	4 (5.19%)
ε33	45 (58.44%)
ε34	16 (20.78%)
ε44	0 (0.0%)
MMSE scores, mean ± SD	**Mean ± SD (*n*** **= 77)**
No cognitive impairment (*n* = 68)	27.91 **±** 1.68
Mild or severe cognitive impairment (*n* = 9)	19.44 **±** 2.35

*APOE* apolipoprotein E

**Fig. 2:**
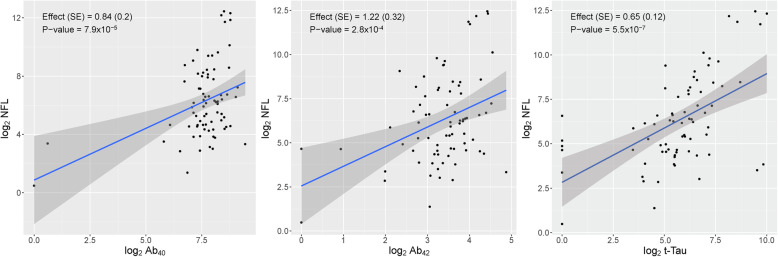
**a**–**c** Regression plots for NfL’s association with AD biomarkers. Higher levels of NfL (*n* = 77) are significantly correlated with higher levels of Aβ_40_ (**a**) *p* = 7.7 × 10^−5^, Aβ_42_ (**b**) *p* = 2.8 × 10^−4^, and t-tau (**c**) *p* = 5.5 × 10^−7^. *p* values were computed from linear regression models after adjusting for diabetes

Further analysis found statistically significant associations between the levels of NfL and Aβ_40_ (*p* value = 7.7 × 10^−5^), Aβ_42_ (*p* = 2.8 × 10^−4^), and t-tau (*p* = 5.5 × 10^−7^) in the vitreous humor, with higher levels of NfL being associated with higher levels of all three biomarkers (Fig. 2a–c and Table 2). All three biomarkers maintained statistical significance after removing values from the analysis that appeared to be outliers (Supplemental Figure 1). Additionally, we found the concentration of NfL in the vitreous has significant associations with inflammatory cytokines and vascular proteins in the vitreous, specifically IL-15 (*p* = 5.3 × 10^−4^), IL-16 (*p* = 2.2 × 10^−4^), MCP-1 (*p* = 4.1 × 10^−4^), VEGFR-1 (*p* = 2.9 × 10^−6^), Vegf-C (*p* = 8.6 × 10^−6^), VCAM-1 (*p* = 5.0 × 10^−4^), Tie-2 (*p* = 6.3 × 10^−4^), and ICAM-1 (*p* = 1.6 × 10^−4^) (Table 3). Many of the biomarkers maintained their significant associations after adjusting for diabetes and after conducting the Bonferroni correction for multiple comparisons. Mean and SD values of NfL and all biomarkers tested in the vitreous humor are presented in Supplemental Table 1, Additional file 1.

**Table 2:** Vitreous NfL levels vs. amyloid beta and tau proteins

***Protein***	***Beta***	***SE***	***p value*** (***n*** = 77)	***p value*** (***a***djusting for DM, ***n*** = 77)
Aβ_40_	0.84	0.20	0.000079	0.0000484
Aβ_42_	1.22	0.32	0.00028	0.00011
t-tau	0.65	0.12	0.00000055	0.00000021
p-tau 181	− 0.30	0.48	0.530	0.646

Associations of NfL with core protein biomarkers for AD in the vitreous humor. As diabetic patients are at higher risk for the development of AD as well as retinal damage, we sought to determine vitreous associations independent of the presence of diabetes, and the significant associations were maintained after adjusting for diabetes

**Table 3:** Association of vitreous NfL levels vs. vitreous levels of inflammatory cytokines and vascular proteins

***Protein***	***Beta***	***SE***	***p value*** (***n*** = 77)	***p value*** (adjusting for DM)
***Pro-inflammatory cytokines***				
IL1α	− 1.43	0.64	0.029	0.044
IL1β	0.59	2.14	0.783	0.846
IL6	0.23	0.11	0.045	0.057
*IL15	1.21	0.33	0.000525	0.00108
*IL16	0.86	0.22	0.00022	0.00044
IL17α	1.01	0.45	0.027	0.027
TNF-α	0.20	0.61	0.748	0.645
IFN-γ	0.32	0.30	0.291	0.289
***Anti-inflammatory cytokines***				
IL4	0.83	0.60	0.17	0.20
IL10	1.22	0.67	0.07	0.053
IL13	0.73	0.55	0.19	0.29
TNFβ	0.99	1.84	0.59	0.778
***Chemokines and inflammatory proteins***				
*MCP-1	0.63	0.17	0.000414	0.000337
3MIP1α	0.19	0.14	0.155	0.153
***Vascular proteins***				
*VEGFR1	0.90	0.17	0.00000285	0.00000292
VEGF-human	0.23	0.10	0.027	0.046
*Vegf-C	0.33	0.07	0.0000086	0.0000139
Vegf-D	0.19	0.08	0.018	0.034
*VCAM-1	0.40	0.11	0.0005	0.0007
*Tie2	0.25	0.07	0.00063	0.0012
*ICAM-1	0.59	0.15	0.00016	0.00028
CRP	0.23	0.10	0.0265	0.0435
SAA	0.14	0.06	0.017	0.0289
bFGF	0.05	0.11	0.616	0.462

Association of NfL with inflammatory cytokines, chemokines, and vascular proteins associated with neurodegenerative disease. The proteins marked with an asterisk (*) maintain statistical significance with NfL after conducting the Bonferroni test for multiple comparisons (0.05/41 analytes with a new *p* = 0.0012). As diabetic patients are at higher risk for the development of AD as well as retinal damage, we sought to determine vitreous associations independent of the presence of diabetes

**Fig. 3:**
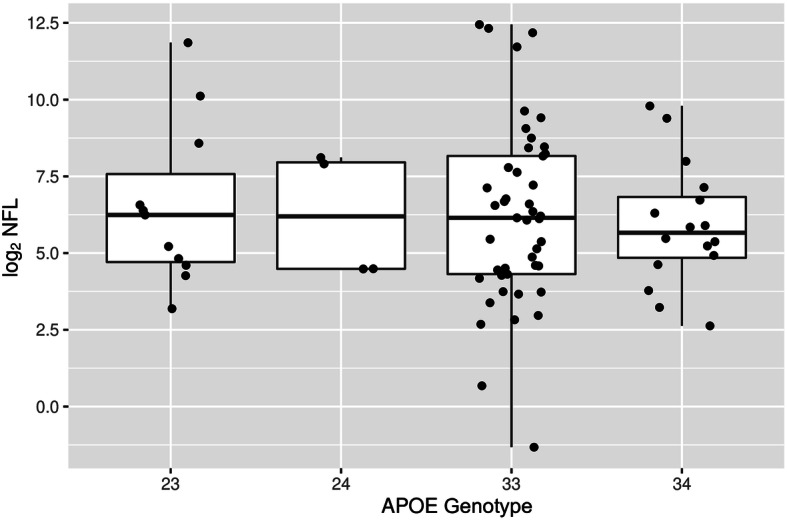
Boxplot of NfL with *APOE* genotype obtained from the subjects’ blood. No significant association is observed between *APOE* alleles 23, 23, 33, and 34, with vitreous NfL levels

Our data also shows that NfL in the vitreous humor was not associated with patients’ clinical eye diagnoses or systemic diseases such as hypertension, diabetes, and hyperlipidemia (Table 4). We also found that NfL was not significantly associated with *APOE* genotypes ε2 and ε4 (Supplemental Table S2, Additional file 2 and Fig. 3), which are the 2 alleles most commonly associated with AD. Furthermore, there were no statistically significant associations of NfL with MMSE scores (Supplemental Table S3, Additional file 3). Additionally, there was no significant correlation between NfL levels in the vitreous humor and patients’ race or ethnicity in our sample population (Supplemental Figures 2 and 3).

**Table 4:** Eye and systemic disease and NfL levels

***Predictor***	***Beta***	***SE***	***t***	***p value***
Macular degeneration	− 1.06	1.38	− 0.77	0.44
Diabetic retinopathy (DR)	0.45	0.61	0.74	0.46
Age-related cataract (ARC)	1.21	1.01	1.19	0.24
ARC + DR	0.22	0.61	0.36	0.72
Cataract surgery	− 0.32	0.68	− 0.47	0.64
Glaucoma	− 0.61	0.90	− 0.68	0.50
Epiretinal membrane	− 1.20	0.74	− 1.62	0.11
Retinal detachment	− 0.29	0.67	− 0.43	0.67
Macular hole	− 0.15	0.84	− 0.17	0.86
Proliferative diabetic retinopathy (stage 4 DR vs. no DR)	0.45	0.62	0.73	0.47
Hypertension	0.28	0.70	0.40	0.69
Diabetes	0.62	0.61	1.01	0.31
Hyperlipidemia	− 0.034	0.63	− 0.05	0.96

No significant association was found between vitreous NfL and the various ophthalmic and systemic diseases in this patient population (*p* > 0.05)

## Discussion

Our study identified and quantified NfL in the vitreous humor of the eye and showed that NfL is positively associated with levels of Aβ_40_, Aβ_42_, and t-tau and other select inflammatory cytokines. Notably, NfL levels were not significantly associated with eye disease, so the levels of NfL in the vitreous humor do not appear to be influenced by the patients’ clinical eye condition(s). In addition, NfL levels were not associated with *APOE* genotypes, and we did not find a significant association with systemic diseases such as diabetes.

Most current studies investigating the role of the eye in neurodegenerative disease, such as AD, focus on retinal biomarkers imaged by optical coherence tomography (OCT). For example, the retinal nerve fiber layer (RNFL) is thinner, the retinal volume is reduced, and the choroidal thickness is reduced in patients with mild cognitive impairment (MCI) and AD compared to cognitively normal (CN) controls [25–38]. A newer and more sophisticated version of the OCT, the optical coherence tomography angiography (OCTA), has shown significant changes to the superficial and deep capillary vascular plexus of the macula and changes to the foveal avascular zone in those with cognitive dysfunction and AD compared to CN controls [39–49]. However, data obtained by OCT/OCTA, while promising, have shown conflicting results and can be confounded by the presence of co-existing eye disease and systemic diseases, such as diabetes, as well as variations in cell layer measurements by different automated platforms [50]. To illustrate this, patients in our study underwent OCT, however, the data was not useful because the patients’ eye diseases, such as diabetic retinopathy, retinal detachment, macular holes, or epiretinal membranes, affected the macular cell layer thickness and volume. Therefore, an additional source of biomarkers, such as one that is quantifiable and protein-based within eye fluid, may potentially offer a more specific marker that may be predictive for neurodegenerative disease.

Current biomarkers for AD comprise 3 categories—amyloid deposition (A), tau pathology (T), and neurodegeneration (N), known as A/T/N biomarkers [51]. “A” signifies changes on amyloid positron emission tomography (PET) imaging and cerebrospinal fluid (CSF) levels of amyloid beta (Aβ1–42); “T” indicates biomarkers of tau, including tau PET or CSF phosphorylated tau (p-tau); and “N” stands for neurodegeneration as reflected by CSF total tau (t-tau), *F*-2-fluoro-2-deoxy-d-glucose PET (FDG-PET), and pathological findings on magnetic resonance imaging (MRI). The clinical utility and diagnostic potential of the core CSF biomarkers for AD (Aβ_40,_ Aβ_42,_ p-tau, and t-tau) are well known and indisputable. However, since access to CSF is more invasive than access to the blood, the utility of testing CSF for the screening of neurodegenerative diseases is limited [52]. Recent data suggest a role for the same protein biomarkers in the blood (plasma or serum), in particular the Aβ42/40 ratio and p-tau181, may be clinically meaningful [53, 54]. Multiple studies have also shown NfL to be significantly associated with more specific biomarkers for AD, particularly tau protein [55–62].

Our study is the first to identify NfL in the vitreous and demonstrate a significant association with Aβ_40_, Aβ_42_, and t-tau; select vitreous cytokines; and proteins, and also the first to establish that NfL is not associated with patients’ local eye condition. However, our results do not show reliable evidence that vitreous NfL levels definitively represent neurodegeneration, and further study is needed to validate NfL in the eye fluid to other established biomarkers of neurodegeneration, such as those found in the CSF or on MRI and PET scan.

The source of elevated NfL in the vitreous humor is unclear. Several pre-clinical studies have shown reduced levels of NfL in the ganglion cell layer of the retina and optic nerve in response to induced injury or ischemia [63–67]. Another study found levels of NfL in the CSF predicted visual outcomes after optic neuritis [68]. Based on this, we can hypothesize that injury, inflammation, or ischemia, from conditions such as glaucoma or optic neuritis which are known to degrade axons in the nerve fiber layer of the retina, results in the local release of NfL from the axons that may diffuse into the vitreous humor through the internal limiting membrane of the retina, leading to elevated NfL in the vitreous. In our cohort, we found no association of elevated NfL levels with eye disease or systemic disease, even in the limited number of patients (13%) who have known local neurodegenerative conditions such as glaucoma [69]. The lack of association with known eye disease indicates another mechanism may be responsible for the elevated levels of NfL in the vitreous.

NfL and t-tau are both known biomarkers for neurodegeneration in the CSF and blood [59]. Since NfL and t-tau are both released from axons as a result of axonal degeneration, the positive correlation we found in the vitreous is consistent with what is seen in the brain and CSF. However, studies have not shown a significant relationship between systemic NfL and systemic amyloid beta proteins (Aβ_40_, Aβ_42_) in the CSF or plasma, most likely due to the difficulty with measuring Aβ, which is derived from the central nervous system, in the blood. The positive association of NfL and Aβ seen in the vitreous humor in our study may be due to a greater ease in testing for Aβ in the vitreous over blood, or possibly an alternative local source of amyloid beta proteins in the eye possibly independent of the amyloid plaques found in the brain [58]. A few pre-clinical mouse studies have indicated that elevated levels of amyloid proteins in the retina and posterior chamber of the eye could be the result of early presymptomatic AD in mice [70, 71].

Our data also showed that NfL was significantly associated with some pro-inflammatory cytokines, chemokines, and vascular injury-related markers in the vitreous humor, and these associations remained significant after adjusting for the presence of diabetes and applying the Bonferroni correction for multiple comparisons (Table 3). As far as we are aware, no prior studies have investigated the association of these inflammatory and vascular proteins with NfL in the CSF, blood, or vitreous humor. But several studies have shown elevated levels of pro-inflammatory cytokines and chemokines in the CSF and blood when the brain experiences neuronal injury, trauma, or neurodegeneration [72–77]. Similarly, microvascular injury biomarkers such as vascular cell adhesion molecule-1 (VCAM-1) and intracellular adhesion molecule-1 (ICAM-1) are released during global and regional cerebral hypoperfusion, a phenomenon associated with neurodegenerative diseases like AD [78]. There is limited research on the diagnostic applicability and clinical utility of these biomarkers, and their significant association with vitreous NfL found in our study indicates further investigation of the interaction of these proteins would increase our understanding of their contribution to pathology.

The clinical utility of obtaining vitreous samples as a possible source of diagnostic testing for AD should be addressed. Obtaining samples of vitreous fluid can be done in 2 ways, through office-based needle aspiration when up to 100 μL can be removed or in the operating room where almost 1 mL of undiluted vitreous can be safely removed. Our study obtained vitreous fluid on patients requiring surgery for a clinical eye condition, and in this setting, it was safe and easy to obtain. However, similar to lumbar puncture for CSF procurement, obtaining vitreous samples for diagnostic testing and screening universally would not be practical nor cost-effective. Investigation of NfL in other eye fluids, such as aqueous humor or tear secretions, could offer a less invasive and more accessible means of fluid collection that could be applied broadly.

### Limitations

This study had some limitations. While levels of NfL were associated with Aβ_40_, Aβ_42_, and t-tau, NfL was not significantly associated with MMSE scores. This may be because testing was done in a non-dementia or non-MCI cohort. MMSE is a crude measurement of cognitive function, and while it is highly specific and valid in the detection of moderate dementia, in mild dementia, it is only 55% sensitive [79]. MMSE was also checked cross-sectionally, and our intent to correlate scores with NfL levels was exploratory; further testing with MMSE longitudinally may shed light on whether NfL in the vitreous is predictive for eventual cognitive decline. Additionally, since no patients in the study carried a diagnosis of AD, validation studies are needed to compare vitreous levels of NfL in patients with MCI or AD to normal controls. This study is the first to investigate the presence of NfL in the vitreous humor by obtaining vitreous samples during surgery. Therefore, a replication sample was not feasible. Furthermore, CSF and blood levels of NfL Aβ40, Aβ42, and t-tau were not obtained, so we were unable to study its correlation and discriminate vitreous levels with CSF and serum levels in order to compare it with established biomarkers of neurodegenerative disease. Further study on this will be very useful in assessing the utility of NfL in the eye fluid. Another limitation was the variance in ocular and systemic co-morbidities among study participants. While our analyses found no significant correlation between NfL and co-existing eye conditions, or systemic conditions such as diabetes, a variety of systemic diseases associated with ocular findings are known to affect cognition. For example, diabetes has been shown to lower cognitive health, increase the risk for development of AD, and release inflammatory cytokines and vascular proteins. On the other hand, diabetic patients are at significant risk for the development of dementia-related to vascular disease and AD, and identifying early markers in the eye may be useful for monitoring this population. Comparing the results of this study to a similar study conducted on a patient population suffering from AD and other types of neurodegenerative disorders will give us a more comprehensive understanding of how this biomarker in the vitreous can contribute as a diagnostic measure.

## Conclusion

In summary, we identified and quantified NfL in the vitreous humor of the eye and significantly associated those levels with proteins such as Aβ_40_, Aβ_42_, and t-tau, and select cytokines and vascular proteins, and established that NfL levels were not influenced by the patients’ clinical eye conditions. These 2 findings are foundational for future studies evaluating the potential utility of NfL in the eye.

## Supplementary information

**Additional file 1: Supplemental Table S1.** Immunoassay Results of all Protein Biomarkers Measured in the Vitreous Humor. The table shows the mean, median, standard deviation and interquartile range of all the proteins measured in the vitreous humor.

**Additional file 2: Supplemental Table S2.** Summary statistics of association between NfL Levels with APOE genotype. The table indicates no significant association was found between vitreous NfL levels APOE genotypes ε2 and ε4.

**Additional file 3: Supplemental Table S3.** Association of NfL Levels with MMSE Scores. The table indicates no significant association was found between vitreous NfL levels and MMSE score; covariates are age, sex and education level.

**Additional file 4: Supplemental Figure S1.** Regression plots for NfL association with AD biomarkers after removal of outliers. Legend: Higher levels of NfL levels are significantly correlated with higher levels of Aβ_40_ (3a, *n*= 74 with *p*=4.9x10^-3^), Aβ_42_ (3b, *n*=73 with *p*=5.4x10^-3^), and t-tau (3c, *n*=68 with *p*=9.0x10^-8^). The significance slope is steeper with a higher *p*-value for t-tau’s association with NfL (3c compared to 2c), after removal of the outliers. *P* values were computed from linear regression models after adjusting for diabetes.

**Additional file 5: Supplemental Figure S2.** Association of NfL Levels with Race. Description of Data: This figure shows no significant association was found between vitreous NfL levels and Race.

**Additional file 6: Supplemental Figure S3.** Association of NfL Levels with Ethnicity. This figure shows no significant association was found between vitreous NfL levels and Ethnicity.

## Data Availability

All data generated or analyzed during this study are included in the published article and its supplementary information files.

## Abbreviations

NfL: Neurofilament light chain
AD: Alzheimer’s disease
Aβ: Amyloid beta
MMSE: Mini-Mental State Examination
APOE: Apolipoprotein E
IL: Interleukin
MCP-1: Monocyte chemoattractant protein-1
VEGFR1: Vascular endothelial growth factor receptor 1
Vegf: Vascular endothelial growth factor
VCAM-1: Vascular cell adhesion protein 1
ICAM-1: Intercellular adhesion molecule 1
MIP1α: Macrophage inflammatory protein-1 alpha
CRP: C-reactive protein
SAA: Serum amyloid A
bFGF: Basic fibroblast growth factor
TNF-α: Tumor necrosis factor alpha
IFN-γ: Interferon gamma
PD: Parkinson’s Disease
CSF: Cerebrospinal fluid
MS: Multiple sclerosis
FTD: Frontotemporal dementia
ALS: Amyotrophic lateral sclerosis
APD: Atypical Parkinsonian disorders
TBI: Traumatic brain injury
ARC: Age-related cataract
R-RD: Rhegmatogenous retinal detachment
MH: Macular hole
ERM: Epiretinal membrane
VH: Vitreous hemorrhage
T-RD: Tractional retinal detachment
DR: Diabetic retinopathy
MGCL: Molecular Genetics Core Laboratory
Simoa: Single-molecule array
MSD: Meso Scale Discovery
OCT: Optical coherence tomography
RNFL: Retinal nerve fiber layer
MCI: Mild cognitive impairment
CN: Cognitively normal
OCTA: Optical coherence tomography angiography
PET: Positron emission tomography
p-tau: Phosphorylated tau
t-tau: Total tau
FDG-PET: *F*-2-fluoro-2-deoxy-d-glucose PET
SE: Standard error
