# Supplementary material for: Neurofilament light chain in the vitreous humor of the eye
Source: Alzheimers Res Ther. 2020 Sep 17;12:111. doi: 10.1186/s13195-020-00677-4 (PMC7500015; doi:10.1186/s13195-020-00677-4)
Supplement: Supplementary file 1 — Additional file 1: Supplemental Table S1. Immunoassay Results of all Protein Biomarkers Measured in the Vitreous Humor. The table shows the mean, median, standard deviation and interquartile range of all the proteins measured in the vitreous humor. [file 13195_2020_677_MOESM1_ESM.docx]

| Supplemental Table S1: Immunoassay results of NfL and all other protein biomarkers in the vitreous humor. | | | | | |
| --- | --- | --- | --- | --- | --- |
| *Biomarker* | ***N*** | ***Mean (pg/mL)*** | ***SD (pg/mL)*** | ***Median (pg/mL)*** | ***IQR (pg/mL)*** |
| NfL | 77 | 432.6671 | 1124.468 | 68.65 | 221.075 |
| Aβ_40_ | 77 | 242.74 | 123.13 | 217.11 | 141.16 |
| Aβ_42_ | 77 | 10.67 | 5.41 | 10.19 | 6.29 |
| t*-*Tau | 75 | 127.91 | 210.47 | 59.79 | 87.19 |
| p*-*tau 181 | 77 | 1.88 | 1.06 | 1.96 | 1.22 |
| IFN*-γ* | 77 | 2.08 | 8.50 | 0.49 | 0.82 |
| IL*-*10 | 77 | 0.31 | 1.02 | 0.12 | 0.09 |
| IL*-*13 | 77 | 1.32 | 1.02 | 1.15 | 0.74 |
| IL*-*1β | 77 | 0.12 | 0.13 | 0.10 | 0.13 |
| IL*-*2 | 77 | 0.37 | 0.22 | 0.34 | 0.20 |
| IL*-*4 | 77 | 0.35 | 1.20 | 0.12 | 0.08 |
| IL*-*6 | 77 | 199.77 | 848.50 | 7.19 | 17.61 |
| IL*-*8 | 77 | 73.56 | 123.93 | 34.08 | 47.54 |
| TNF*-α* | 77 | 0.52 | 0.94 | 0.26 | 0.23 |
| IL*-*12 p*-*70 | 77 | 370.88 | 373.72 | 190.53 | 768.34 |
| IL*-*15 | 69 | 5.76 | 4.76 | 4.59 | 4.09 |
| IL*-*16 | 69 | 27.66 | 34.99 | 14.87 | 19.65 |
| IL*-*17A | 69 | 1.13 | 4.57 | 0.52 | 0.35 |
| IL*-*5 | 69 | 0.35 | 0.45 | 0.23 | 0.20 |
| IL*-*7 | 69 | 20.20 | 12.11 | 18.54 | 15.67 |
| TNF*-*β | 69 | 0.13 | 0.15 | 0.09 | 0.15 |
| VEGF | 69 | 134.33 | 462.98 | 10.30 | 94.04 |
| IL*-*1α | 69 | 0.42 | 1.24 | 0.19 | 0.29 |
| bFGF | 77 | 186.45 | 663.17 | 5.73 | 23.25 |
| *VEGFR1* | 77 | 49652.09 | 30788.15 | 40065.04 | 40119.06 |
| Tie2 | 77 | 259.96 | 593.52 | 11.87 | 149.89 |
| VEGF *(*human*)* | 77 | 39.33 | 150.94 | 0.00 | 33.07 |
| Vegf*-*C | 77 | 127.55 | 268.57 | 0.00 | 130.96 |
| Vegf*-*D | 77 | 243.47 | 369.48 | 87.26 | 362.21 |
| CRP | 77 | 2915184.56 | 4914452.40 | 645009.60 | 3411701.02 |
| ICAM1 | 77 | 381365.14 | 416245.28 | 272408.70 | 389874.08 |
| SAA | 77 | 2203851.92 | 6090667.99 | 142280.41 | 864854.69 |
| VCAM1 | 77 | 240803.89 | 284394.14 | 145385.12 | 272275.37 |
| Eotaxin | 77 | 92.74 | 84.30 | 78.05 | 96.89 |
| Eotaxin*-*3 | 77 | 26.06 | 55.50 | 8.42 | 27.74 |
| IP*-*10 | 77 | 6147.55 | 11886.94 | 2605.11 | 3038.89 |
| MCP1 | 77 | 6812.10 | 7416.16 | 4909.50 | 5982.87 |
| MCP4 | 77 | 18.36 | 23.69 | 0.00 | 31.52 |
| MIP1a | 77 | 55.07 | 105.59 | 33.99 | 34.22 |
| MIP1*b* | 77 | 87.43 | 97.37 | 59.63 | 64.94 |
| TARC | 77 | 23.96 | 48.00 | 10.63 | 17.60 |
| The table shows the mean, median, standard deviation and interquartile range of all the proteins.  Abbreviations: Aβ=amyloid-beta; t-tau=total tau; p-tau=phosphorylated tau; IL=interleukins; TNF-α=tumor necrosis factor alpha; IFN-γ=interferon gamma; MCP-1=monocyte chemoattractant protein-1; MIP1α=macrophage inflammatory protein- 1 alpha; VEGFR1=vascular endothelial growth factor receptor 1; VEGF=vascular endothelial growth factor; VCAM-1=vascular cell adhesion molecule 1; ICAM-1=intracellular adhesion molecule 1; CRP=C-reactive protein; SAA=serum amyloid A; bFGF=basic fibroblast growth factor; IP-10=interferon gamma-induced protein 10; TARC=thymus and activation regulated chemokine; SD= standard deviation; IQR=interquartile range. | | | | | |
