# Supplementary material for: Neurofilament light chain in the vitreous humor of the eye
Source: Alzheimers Res Ther. 2020 Sep 17;12:111. doi: 10.1186/s13195-020-00677-4 (PMC7500015; doi:10.1186/s13195-020-00677-4)
Supplement: Supplementary file 2 — Additional file 2: Supplemental Table S2. Summary statistics of association between NfL Levels with APOE genotype. The table indicates no significant association was found between vitreous NfL levels APOE genotypes ε2 and ε4. [file 13195_2020_677_MOESM2_ESM.docx]

| Supplemental Table S2: Summary statistics of association between NfL Levels with APOE genotype. | | | | |
| --- | --- | --- | --- | --- |
| *Outcome* | ***Predictor*** | ***Beta*** | ***SE*** | ***p*** |
| log_2_ NFL | APOE ε2 | -0.027 | 0.785 | 0.973 |
| log_2_ NFL | APOE ε4 | -0.357 | 0.742 | 0.632 |
| The table indicates no significant association was found between vitreous NfL levels APOE genotypes ε2 and ε4.  Abbreviations: NfL=neurofilament light chain, APOE=apolipoprotein E. | | | | |
