# Supplementary material for: Neurofilament light chain in the vitreous humor of the eye
Source: Alzheimers Res Ther. 2020 Sep 17;12:111. doi: 10.1186/s13195-020-00677-4 (PMC7500015; doi:10.1186/s13195-020-00677-4)
Supplement: Supplementary file 3 — Additional file 3: Supplemental Table S3. Association of NfL Levels with MMSE Scores. The table indicates no significant association was found between vitreous NfL levels and MMSE score; covariates are age, sex and education level. [file 13195_2020_677_MOESM3_ESM.docx]

| Supplemental Table S3: Association of NfL Levels with MMSE Scores | | | | | |
| --- | --- | --- | --- | --- | --- |
| *Outcome* | ***Predictor*** | ***Covariate*** | ***Beta*** | ***SE*** | ***p-value*** |
| MMSE score† | log_2_NfL | **‡**Education (ordinal) | 0.09 | 0.14 | 0.52 |
| MMSE score | log_2_NfL | **‡**Education (categorical) | 0.09 | 0.12 | 0.49 |
| The table indicates no significant association was found between vitreous NfL levels and MMSE score; covariates are age, sex and education level.  †score refers to the MMSE score ranging 0 to 30.  ‡Education level was evaluated as both an ordinal and categorical variable.  Abbreviations: MMSE=Mini-mental State Examination, NfL=Neurofilament Light Chain | | | | | |
