# Supplementary figures and images for: Neurofilament light chain in the vitreous humor of the eye
Source: Alzheimers Res Ther. 2020 Sep 17;12:111. doi: 10.1186/s13195-020-00677-4 (PMC7500015; doi:10.1186/s13195-020-00677-4)

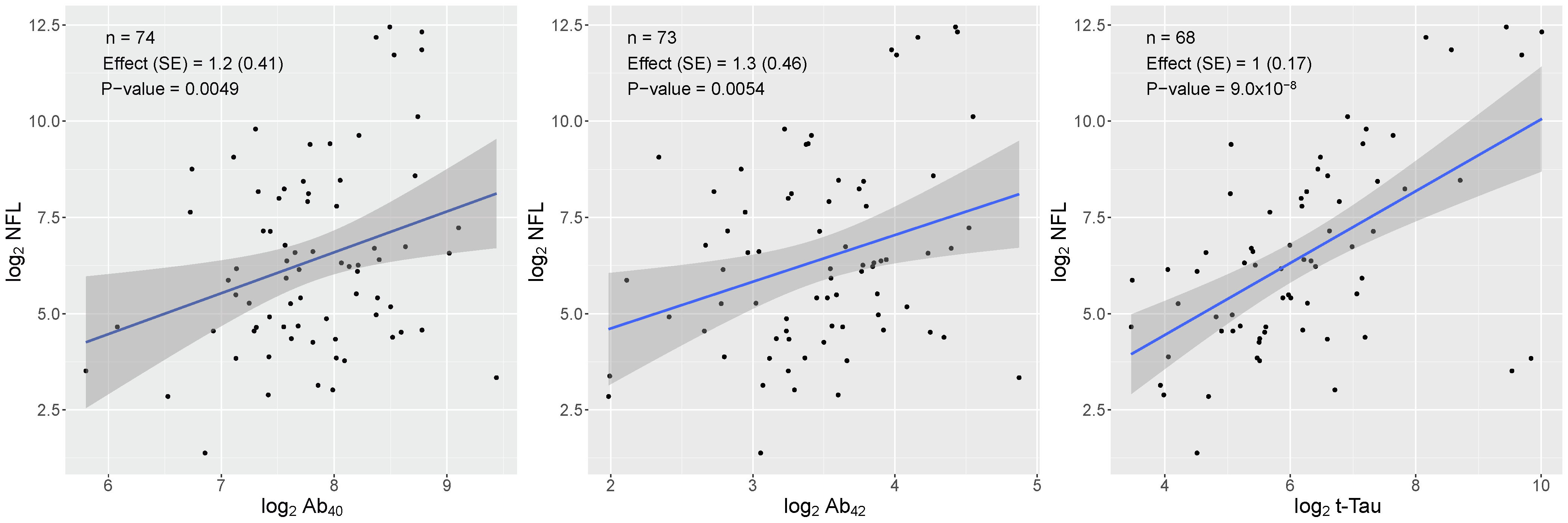

Supplement: Supplementary file 4 — Additional file 4: Supplemental Figure S1. Regression plots for NfL association with AD biomarkers after removal of outliers. Legend: Higher levels of NfL levels are significantly correlated with higher levels of Aβ40 (3a, n= 74 with p=4.9x10^-3^), Aβ42 (3b, n=73 with p=5.4x10^-3^), and t-tau (3c, n=68 with p=9.0x10^-8^). The significance slope is steeper with a higher p-value for t-tau’s association with NfL (3c compared to 2c), after removal of the outliers. P values were computed from linear regression models after adjusting for diabetes. [file 13195_2020_677_MOESM4_ESM.tif]

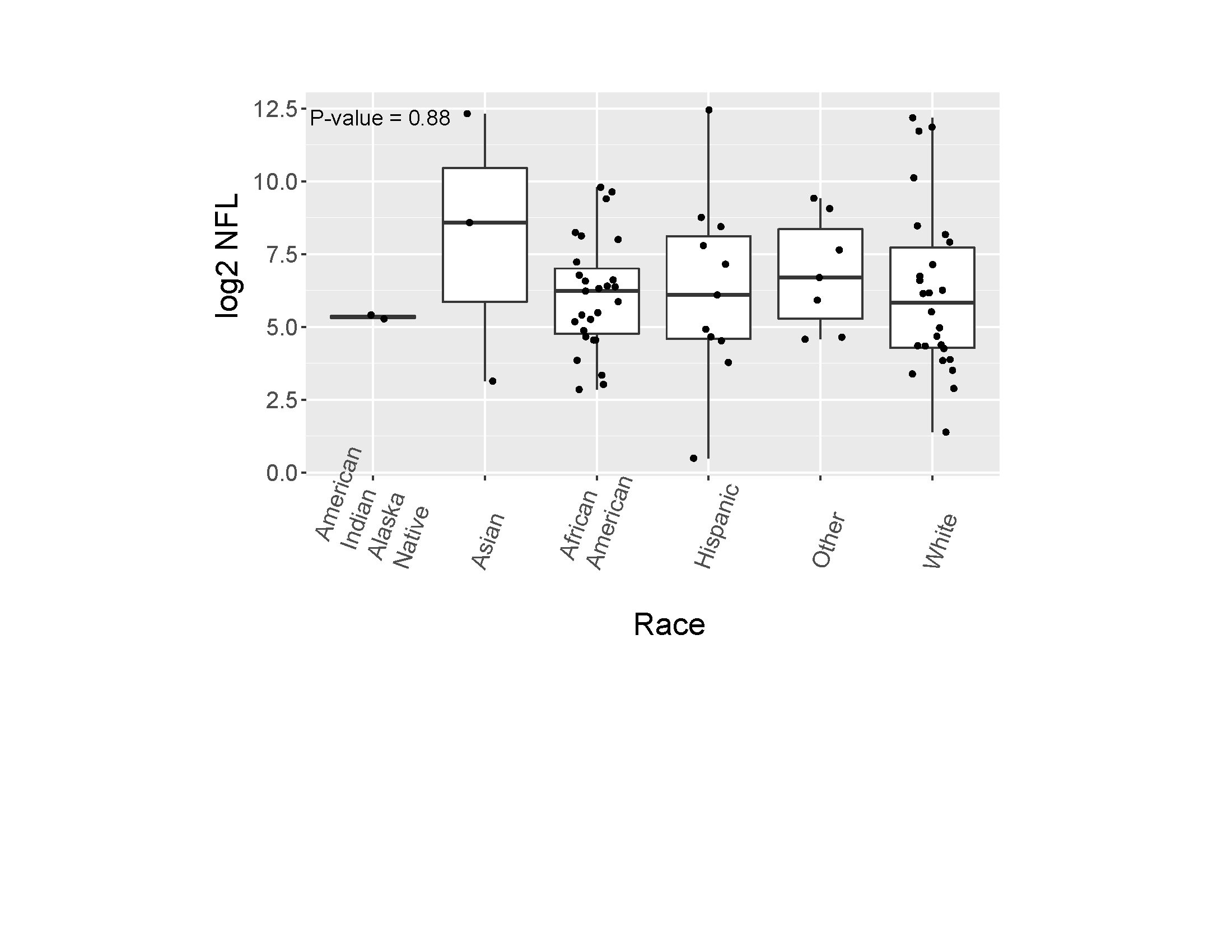

Supplement: Supplementary file 5 — Additional file 5: Supplemental Figure S2. Association of NfL Levels with Race. Description of Data: This figure shows no significant association was found between vitreous NfL levels and Race. [file 13195_2020_677_MOESM5_ESM.tiff]

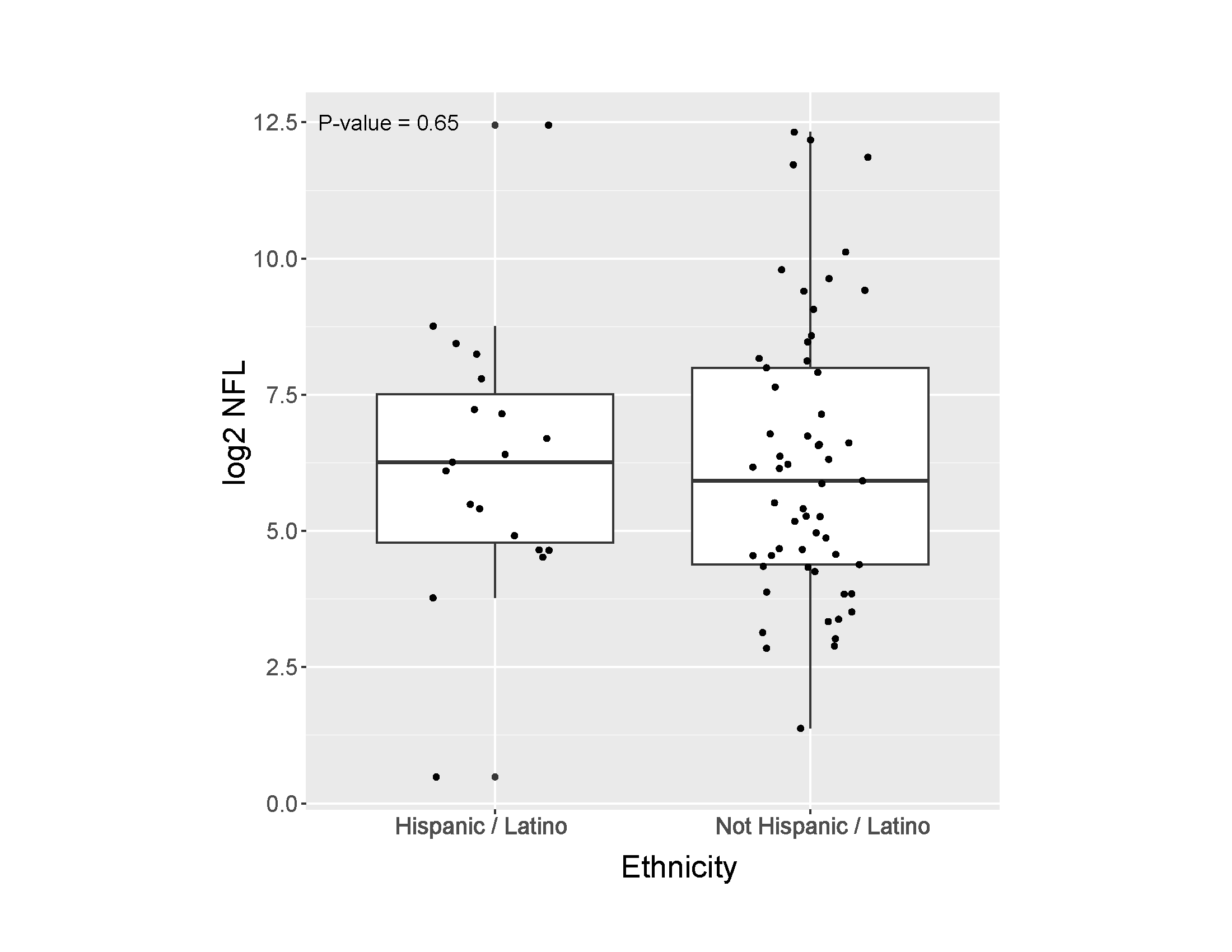

Supplement: Supplementary file 6 — Additional file 6: Supplemental Figure S3. Association of NfL Levels with Ethnicity. This figure shows no significant association was found between vitreous NfL levels and Ethnicity. [file 13195_2020_677_MOESM6_ESM.tiff]
